# Emerging threat of multidrug resistant bugs – *Acinetobacter calcoaceticus baumannii* complex and Methicillin resistant *Staphylococcus aureus*

**DOI:** 10.1186/1756-0500-6-98

**Published:** 2013-03-15

**Authors:** Shyam Kumar Mishra, Basista Prasad Rijal, Bharat Mani Pokhrel

**Affiliations:** 1 Department of Microbiology, Institute of Medicine, Tribhuvan University, Kathmandu, Nepal

**Keywords:** *Acinetobacter* spp., MDR, MRSA, ESBL

## Abstract

**Background:**

Infections caused by bacteria such as multidrug resistant (MDR) *Acinetobacter* spp. and methicillin-resistant *Staphylococcus aureus* (MRSA) constitute a worldwide pandemic. Without gathering information about these strains, we cannot reduce the morbidity and mortality due to infections caused by these notorious bugs.

**Methods:**

This study was conducted to identify the status of MDR *Acinetobacter* spp. and MRSA in a tertiary care centre of Nepal. Sputum, endotracheal aspirate and bronchial washing specimens were collected and processed from patients suspected of lower respiratory tract infection following standard microbiological methods recommended by the American Society for Microbiology (ASM). Double disk synergy test method was employed for the detection of extended-spectrum beta-lactamase (ESBL) in *Acinetobacter* isolates. Methicillin resistance in *S. aureus* was confirmed by using cefoxitin and oxacillin disks.

**Results:**

Different genomespecies of *Acinetobacter* were isolated; these consisted of *Acinetobacter calcoaceticus baumannii* complex and *A. lwoffii*. Around 95% of *Acinetobacter* isolates were MDR, while 12.9% were ESBL-producer. Of the total 33 isolates of *S. aureus*, 26 (78.8%) were MDR and 14 (42.4%) were methicillin resistant.

**Conclusions:**

A large number of MDR *Acinetobacter* spp. and MRSA has been noted in this study. The condition is worsened by the emergence of ESBL producing *Acinetobacter* spp. Hence, judicious use of antimicrobials is mandatory in clinical settings. Moreover, there should be vigilant surveillance of resistant clones in laboratories.

## Background

Worldwide emergence of multidrug resistance among gram-negative and gram-positive bacteria has resulted in a confounding scene in treatment modalities. Bacteria like *Acinetobacter calcoaceticus baumannii* (Acb) complex and methicillin resistant *Staphylococcus aureus* (MRSA) have created a dilemma regarding the appropriate antibiotic therapy to use against them. Infections caused by these bugs are often difficult to treat. Further, these bacteria survive for a long time in hospital environment, with enhanced opportunities for transmission between patients [1]. It is noteworthy that hospital-acquired pneumonia (HAP) developing ≥ 5 days after hospitalization (late-onset) is often caused by aerobic gram-negative bacilli (e.g., *Acinetobacter* spp.) or MRSA [2].

MRSA is one of the most important nosocomial pathogens worldwide, but recently it is increasingly identified as the etiological agent of infections acquired in community. Molecular epidemiological studies indicate that community associated (CA)-MRSA and healthcare associated (HA)-MRSA may have distinctive phenotypic and genetic features [3]. Traditionally, CA-MRSA are attributed with characteristics, such as smaller staphylococcal chromosomal cassette (SCC)*mec* cassettes – types IV and V – and a more restricted resistance pattern to antibiotics other than β-lactams than HA-MRSA. However, recently, a bidirectional crossing of borders between HA- and CA-associated infections is occurring [4]. These days, MRSA infections acquired outside of the hospital setting have been increasingly reported. The resistance of MRSA against various antimicrobials is globally increasing at an alarming rate. As a result, treatment of MRSA infections has become more challenging. This is a disturbing revelation and a major concern among health care professionals.

Studies of multidrug resistant (MDR) bacterial isolates like *Acb* complex and MRSA are crucial not only for the proper management of infections caused by them, but also for the prevention of the dissemination of such strains in the community and in hospitals. We undertook this study to find out the current trend of drug resistant *Acinetobacter* spp. and MRSA isolates in clinical samples from patients suspected of lower respiratory tract infections (LRTIs). Additionally, we aimed to determine the prevalence of ESBL in *Acinetobacter* and to compare the role of oxacillin and cefoxitin discs in detecting methicillin resistance in *S. aureus*.

## Methods

A hospital-based prospective study was carried out in the Bacteriology Laboratory of Tribhuvan University Teaching Hospital (TUTH) in Kathmandu, Nepal over a period of six months. During the study period, 1120 specimens were processed.

### Inclusion criteria

Specimens obtained from the lower respiratory tract [sputum, endotracheal (ET) secretion and bronchial washing] were included for culture and sensitivity test. Only those specimens which met the criteria recommended by the American Society for Microbiology (ASM) [5] were selected for further processing.

### Culture of the specimen and identification of isolated organisms

The specimens were cultured on Chocolate agar (CHA), 5% Sheep Blood agar (BA) and MacConkey agar (MA) (Oxoid, UK) plates. The CHA plates were incubated in a CO_2_ incubator (10% CO_2_) at 37°C for 24 hours. The BA and MA plates were incubated at 37°C for 24 hours in an aerobic atmosphere. Identification of significant isolates was done following standard microbiological techniques [5]. A purity plate was employed to ensure that the inoculum used for the biochemical tests was pure.

Duplicate isolates from the same patient were excluded from the study.

### Antibiotic susceptibility testing

The antibiotic sensitivity test of the pathogens isolated from the clinical specimen was done in Mueller Hinton agar (MHA) (Oxoid, UK) by Kirby-Bauer method, as recommended by Clinical and Laboratory Standards Institute (CLSI) [6].

For disk susceptibility testing, penicillin, amoxycillin, oxacillin, ciprofloxacin, cotrimoxazole, cephalexin, gentamicin, erythromycin, clindamycin, teicoplanin, vancomycin, amoxicillin-clavulanate, cloxacillin, linezolid and cefoxitin were used for *S. aureus*. Likewise, ciprofloxacin, cotrimoxazole, gentamicin, amikacin, cefotaxime, ceftriaxone, ceftazidime, cefepime, amoxicillin-clavulanate, piperacillin, piperacillin-tazobactam, tigecycline,cefoperazone-sulbactam, meropenem and imipenem were used for *Acinetobacter* spp. The initial screen test for the production of ESBL was performed by using ceftriaxone (CRO) (30 μg), ceftazidime (CAZ) (30 μg) and cefotaxime (CTX) (30 μg) disks (Oxoid, UK). If the zone of inhibition (ZOI) was ≤ 25 mm for CRO, ≤ 22 mm for CAZ and/or ≤ 27 mm for CTX, the isolate was considered a potential ESBL-producer as recommended by CLSI [6]. Isolates those that were suspected as ESBL-producer by screen test were tested further by double disk synergy test (DDST). In DDST method, amoxicillin-clavulanic acid (AMC) disk (20/10 μg) was placed at the center and disks containing the 30 μg of CAZ, CTX and CRO were placed separately beside 15 mm distance (edge to edge), away from the central disk, in a horizontal manner [7]. Any enhancement of the ZOI between the disks (either of the cephalosporin disks and clavulanic acid containing disk) indicated the presence of ESBL.

In this study, if the isolates were resistant to at least three classes of first line antimicrobial agents, they were regarded as MDR [8]. Inducible macrolide-lincosamide-streptogramin B (iMLS_B_) resistance was detected in *S. aureus* by Disk approximation test placing a 2 microgram clindamycin disk 15 mm away from the edge of a 15 microgram erythromycin disk on a MHA plate. Following incubation, organisms that showed flattening of the clindamycin zone adjacent to the erythromycin disk (referred to as a "D" zone) were considered to exhibit inducible clindamycin resistance [6].

To test for MRSA, a 1 μg oxacillin disk and a 30 μg cefoxitin disk were used, and the plates were incubated at 35°C. Plates containing oxacillin disk were read following a 24 hour incubation period. The diameter of the ZOI of growth was recorded and interpreted as susceptible or resistant by the criteria of CLSI. *S. aureus* isolates were deemed methicillin resistant when the ZOI was ≤10 mm with the oxacillin disk test or ≤21 mm with the cefoxitin disk method [6].

Control strains of *E. coli* ATCC 25922 and *S. aureus* ATCC 25923 were used for the standardization of the antibiotic sensitivity test. For ESBL test standardization, *E. coli* ATCC 25922 and *K. pneumoniae* ATCC 700603 were used as negative and positive controls respectively. *S. aureus* strains ATCC 25923 and ATCC 43300 were used as negative and positive controls respectively for MRSA test standardization.

### Ethical consideration

Permission was obtained from the Institutional Review Board at the Institute of Medicine, Tribhuvan University, Kathmandu. Written informed consent for participation in the study was obtained from participants or where participants were children, the consent was obtained from their parent or guardian.

### Data processing and analysis

Data were analyzed using Microsoft Excel 2007 and represented as frequency distribution and percentage.

## Results

### Number of specimens and result pattern

Among the total processed specimens (n=1120), 497 showed significant growth (44.38%). Of all the isolates, *Acinetobacter* spp., the organism of our interest, was the fourth most common bacterial isolate (11.23%). *Acinetobacter* spp. comprised of both *Acb* complex and *A. lwoffii*. Likewise, *S. aureus* was the seventh most common significant bacteria (6.19%) to cause LRTIs. A majority of *Acinetobacter* spp. (88.3%) *and S. aureus* (75.76%) were isolated from inpatients.

### Antimicrobial susceptibility test result of *Acinetobacter* spp

Nearly 58%, 38.7% and 35% of *Acinetobacter* isolates showed resistance to piperacillin-tazobactam, tigecycline and imipenem respectively (Table 1). Approximately 95% of *Acinetobacter* isolates were MDR, while 12.9% were ESBL-producer (Data not shown in table).

**Table 1:** **Antimicrobial susceptibility test result of *Acinetobacter *spp. (n=62)**

**Antibiotics**	**Resistant (%)**
Ciprofloxacin	64.5
Cotrimoxazole	75.8
Gentamicin	62.9
Amikacin	54.8
Ceftriaxone	87.1
Cefotaxime	88.7
Ceftazidime	82.3
Cefepime	85.5
Amoxycillin-Clavulanate	88.7
Piperacillin	77.4
Piperacillin-Tazobactam	58.0
Cefoperazone-Sulbactam	54.8
Meropenem	50.0
Imipenem	35.5
Tigecycline	38.7

### Antimicrobial susceptibility test result of *S. aureus*

Most of the commonly used oral antibiotics were not effective against *S. aureus*. Only vancomycin, teicoplanin and linezolid were found to be the most effective antibiotics (Table 2).

**Table 2:** **Antimicrobial susceptibility test result of *S. aureus *(n=33)**

**Antibiotics**	**Resistant (%)**
Penicillin	93.9
Amoxycillin	90.9
Ciprofloxacin	69.7
Cotrimoxazole	72.7
Cephalexin	84.6
Gentamicin	54.5
Amoxycillin-clavulanate	69.7
Erythromycin	63.6
Clindamycin	45.5
Oxacillin	36.4
Cefoxitin	42.4
Cloxacillin	42.4
Vancomycin	0.0
Teicoplanin	0.0
Linezolid	0.0

### MRSA and Inducible clindamycin resistance

Of the total 33 isolates of *S. aureus*, 14 (42.4%) were methicillin-resistant. Incidence of inducible clindamycin resistance was also seen (3.0%).

### Number of MDR bacterial isolates

Nearly 95% of *Acinetobacter* spp. and 78.8% of *S. aureus* isolates were MDR.

### Distribution of Acinetobacter and MRSA

*Acinetobacter* spp. and MRSA were more common in intensive care units (ICUs) (Table 3).

**Table 3:** **Wardwise distribution of ESBL- producing *Acinetobacter *spp. and MRSA**

**Wards**	***Acinetobacter *spp.**	**MRSA**
	**No.**	**No.**
Surgical	9	2
Medical	14	4
Cardiac care unit (CCU)	5	1
ICU	23	6

### Comparison of Cefoxitin and Oxacillin methods for MRSA detection

Among the 33 *S. aureus* isolates subjected to the cefoxitin (30 μg) and oxacillin (1 μg) disk methods, the former detected 14 (42.4%) to be MRSA and the latter detected 12 (36.4%). The cefoxitin disk method detected more MRSA (*P*>0.05).

## Discussion

This study was conducted among the patients attending TUTH, Kathmandu to find out the bacteriology of LRTIs with special reference to *Acinetobacter* spp. and MRSA.

A higher prevalence of *Acinetobacter* spp. was encountered in this study. *Acinetobacter* spp. consisted of *Acb* complex (10.85%) and *A. lwoffii* (0.38%). A prevalence study carried out a decade ago at TUTH showed the growth of *Acinetobacter* spp. to be 1.19% of the total number of cases [9]. Another study in the same setting in 2004 showed the increment in growth of these bacteria by twofold [10]. Likewise, the present finding on the prevalence of *Acinetobacter* spp. is higher than reported 3.9% of LRTI cases in Western Nepal [11]. These findings show an increasing trend in the prevalence of *Acinetobacter* spp.

In this study, out of total 62 *Acinetobacter* isolates, 51 (85.0%) were from inpatients and 48 (77.42%) were from elderly patients. The infection caused by *Acinetobacter* most frequently involved the respiratory tract of intubated patients. Studies have shown that *Acinetobacter* pneumonia is more common in critically ill patients in Asian (range 4-44%) and European hospitals (0-35%); however, it has a low incidence in United States hospitals (6-11%) [12].

Fluoroquinolones have replaced other quinolones over the last two decades. They are now widely used in clinical medicine as broad-spectrum antimicrobial agents. As a result, fluoroquinolone resistance is increasing rapidly [13]. In our study, ciprofloxacin resistance was found in 64.52% of *Acinetobacter* spp. isolates. Higher resistance (72.9% and (>90%) to this antibiotic has been reported in India by Joshi et al. [13] and Mezzatesta et al. [14]. *Acinetobacter* spp. are known to harbour resistance plasmids (R-plasmids), and therefore often act as a reservoir of multidrug-resistance [15]. These pathogens can mutate to acquire quinolone resistance by either target alteration that modifies DNA gyrase (DNA topoisomerase II), or diminished drug accumulation by modifying porin system and efflux pump [16].

Out of 62 *Acinetobacter* isolates, 59 (95.16%) were MDR and 88.14% of the MDR *Acinetobacter* isolates were from inpatients. This startling rate of MDR *Acinetobacter* underscores the need for a cogent step in treatment options. Higgins et al. [16] suggested that sulbactam had better activity over clavulanate and tazobactam and it might represent an alternative treatment option for infections due to the MDR *Acb* complex. Ling et al. [17] also reported a higher sensitivity of *Acb* complex against cefoperazone-sulbactam than piperacillin-tazobactam in Shanghai (91% vs. 29%) and Hong-Kong (95% vs. 81%). Complying with the previous studies, this study also showed cefoperazone-sulbactam had a comparatively lower resistance rate (54.84%) than piperacillin-tazobactam (58.07%) and amoxycillin-clavulanate (88.71%); however, the difference is not significant for the former. Tigecycline, a glycylcycline, was found to be a promising antibiotic. However, caution should be used in considering tigecycline treatment for *A. baumannii* infection in sites where drug levels may be suboptimal, such as the bloodstream. Development of resistance to tigecycline has been reported recently [18].This demands prudent use of this novel antibiotic in treatment modalities.

The resistance of *Acinetobacter* spp. towards the carbapenems is much higher in this study (50% for meropenem and 35.48% for imipenem) as compared to different studies in Indian hospitals, viz., All India Institute of Medical Sciences (34.7% for meropenem and 27.2% for imipenem) [19], and St. John’s Hospital (14.0% for meropenem) [20].

The decreased susceptibility of gram-negative isolates towards the third and fourth generation cephalosporins (26.79% to 28.57%) could be attributable to ESBL or AmpC beta-lactamase production. We found that 12.9% of *Acb* complex were ESBL-producer. However, we did not perform test for AmpC beta lactamase. Due to the increase in ESBL producing strains, there has been increased use of beta-lactam/ beta-lactamase inhibitor combinations, monobactams and carbapenems. However, the last few years have witnessed an increasing resistance to these drugs as well. In India, 22.2% and 17.3% of ESBL producers showed resistance to meropenem and imipenem respectively [19]. Though this reported finding is slightly higher than the present study finding, the possibility of the emergence of carbapenem-resistant isolates cannot be denied.

Medical wards and ICUs comprised the major domicile of ESBL producers. Third generation cephalosporin, such as ceftriaxone, cefotaxime and ceftazidime, are highly used in ICUs, even in our setting. Therefore, the resistance observed here may have appeared under the selective influence of the extensive usage of these antibiotics. In any nosocomial setting, carbapenems are used as the last resort for treatment of MDR *Acinetobacter* infections. However, over the last 15 years, acquired resistance to this life saving antimicrobial has been increasingly reported [21]. The pan-resistant gram-negative ESBL producing isolates were sensitive to polymyxin B and colistin sulphate. Because no fundamentally new anti-infective drugs are currently available, it compelled us to re-evaluate the ‘old drugs’ and, fortunately, they proved to be as effective in this study as in other studies [22].

Apart from drug resistance in gram-negative bacteria, a worldwide increase in antibiotic resistance has been observed among gram-positive isolates. Today the concern of MRSA has reached the pinnacle. It is noteworthy that MRSA can cause both community- and hospital- acquired infections. However, there are only a few reports depicting the situation of MRSA among LRTI isolates in Nepal. In this study, out of total 33 *S. aureus* isolates, 14 (42.42%) were identified as MRSA. Nearly 93% of MRSA were isolated from inpatients (*P*<0.04). Considering the specimenwise distribution, 78.59% of the MRSA were isolated from sputum specimens, while the rest were from ET secretion. In eastern Nepal, Kumari et al. found that 60% of *S. aureus* isolates from respiratory tract specimens were methicillin resistant. Though the finding of the present study showed less prevalence of MRSA as compared to Kumari et al. [23], it is much higher than that of 13.01% reported by Pokhrel et al. [24] from TUTH in 1993 and 15.4% by Subedi et al. [25] from Western Nepal in 2005.

Prior antibiotic use is the most common risk factor for colonization and infection with MRSA. Other risk factors for pneumonia due to MRSA include use of corticosteroids and chronic obstructive pulmonary disease. Mortality rates are higher in patients with pneumonia caused by MRSA than in those with pneumonia caused by methicillin susceptible *S. aureus*[26]. This heightened mortality likely reflects more serious comorbidities rather than differences in the virulence of the organisms [27]. In Japan, MRSA isolates from sputum specimens dramatically increased from 0% in 1985 to 51.4% in 1988. Moreover, 7 out of 13 cases of bronchopulmonary infections due to MRSA died [28]. The SENTRY antimicrobial surveillance (1997–1999) program found that the prevalence of MRSA in hospitals was very high in Singapore (62.9%), Taiwan (61.1%) and Portugal (54.4%) [29].

This study demonstrated that the overall sensitivity pattern of *S. aureus* isolates, with the exception to clindamycin, linezolid, teicoplanin, and vancomycin, other commonly prescribed antibiotics, had their spectrum of action much below 70%. Nearly 54.54% of the isolates were sensitive to clindamycin and 100% to linezolid, teicoplanin and vancomycin. Agents like erythromycin, ciprofloxacin and cotrimoxazole were effective in only 36.36%, 30.30%, and 27.27% cases, respectively. Resistance to penicillin was found in 93.94% of the *S. aureus* isolates. This finding complies with that of Dar et al. [30], who showed 92.0% of the *S. aureus* were resistant to penicillin.

In this study, a very low sensitivity of MRSA strains towards ciprofloxacin is probably due to the indiscriminate empirical use of these drugs because of their low cost and ease of availability in our country. High resistance to ciprofloxacin has also been observed by Subedi et al. [25]. The susceptibility towards erythromycin was also lower in this study when compared with that of Kumari et al. (28.69%) [23]. Clindamycin is one of the drugs of choice in MRSA infections. Macrolide-resistant isolates of *S. aureus* may have constitutive or inducible resistance to clindamycin (methylation of the 23S rRNA encoded by the *erm* gene also referred to as MLS_B_ i.e., Macrolide, Lincosamide and type B Streptogramin resistance) or may be resistant only to macrolides (efflux mechanism encoded by the *msr*A gene) [6]. In this study, one erythromycin inducible clindamycin resistant case in an MRSA strain was also observed by D test. Vancomycin is used to treat MDR infections. In this study, no isolate was found to be vancomycin resistant. Vancomycin seemed to be the only option as the last resort antibiotic for MDR staphylococcal infection.

Two different methods were employed for the detection of MRSA. The cefoxitin disk method was better than the oxacillin disk method (*P*>0.05), as the former detected all 14 MRSA cases while the latter missed two cases. According to CLSI [6], the cefoxitin disk test is comparable to the oxacillin disk test for the prediction of *mec*A-mediated resistance to oxacillin. The cefoxitin disk test is easier to read and thus is the preferred method. Besides, cefoxitin is an inducer of the *mec*A gene.

## Conclusions

Notorious bugs such as MDR *Acinetobacter* spp. and MRSA have emerged. Although combination drugs and carbapenems are relatively more effective against *Acinetobacter* spp., there is a confounding scene in antibiotic armamentarium due to reports on resistance of these bugs against the last resort antibiotics. Thus, there is a great threat of dissemination of such resistant clones in the community as well as in hospital settings. It is mandatory to control this situation before it leads to a disaster. Hospital infection control program should be effectively implemented for the management of infected patients and to breach the transmission of such organisms to other patients, health care workers, and to the community.

## Abbreviations

MDR: Multidrug resistant; MRSA: Methicillin resistant *Staphylococcus aureus*; ASM: American Society for Microbiology; ESBL: Extended-spectrum beta-lactamase; HAP: Hospital-acquired pneumonia; LRTI: Lower respiratory tract infection; TUTH: Tribhuvan University Teaching Hospital; ET: Endotracheal; MHA: Mueller Hinton agar; CLSI: Clinical and Laboratory Standard Institute; CA-MRSA: Community associated Methicillin resistant *Staphylococcus aureus*; HA-MRSA: Healthcare associated Methicillin resistant *Staphylococcus aureus*; iMLSB: Inducible macrolide lincosamide and streptogramin B; DDST: Double disk synergy test; ZOI: Zone of inhibition; ATCC: American type culture collection; Fig.: Figure; No.: Number; ICU: Intensive care unit; CCU: Cardiac care unit

## Competing interests

The authors declare that they have no competing interests.

## Authors’ contributions

SKM carried out the study, analyzed data and prepared the manuscript. BPR and BMP designed the study and supervised the work and the manuscript. All authors have read and approved the manuscript.

## References

[B1] Bergogne-Berezin E Towner KJ Acinetobacter species as nosocomial pathogens: microbiological, clinical, and epidemiological features Clin Micro Rev 1999 9 148 165 8964033 10.1128/cmr.9.2.148 PMC172888

[B2] Cohen J Powderly WG Community-acquired pneumonia Infectious disease 2004 1 2 Mosby: Elsevier 369 380

[B3] Mammina C Calà C Bonura C Carlo PD Aleo A Fasciana T Giammanco A EPI-MRSA Working Group Polyclonal non multiresistant Staphylococcus aureus isolates from clinical cases of infection occurring in Palermo, Italy, during a one-year surveillance period Ann Clin Microbiol Antimicrob 2012 11 17 10.1186/1476-0711-11-17 22713430 PMC3473248

[B4] Lesosky M McGeer A Simor A Green K Low DE Raboud J Effect of patterns of transferring patients among healthcare institutions on rates of nosocomial methicillin-resistant Staphylococcus aureus transmission: a Monte Carlo simulation Infect Contr Hosp Epidemiol 2011 32 136 147 10.1086/657945 21460468

[B5] HD I Clinical Microbiology Procedures Handbook 2004 2 Washington DC: ASM press

[B6] Clinical and Laboratory Standards Institute Performance standards for antimicrobial susceptibility testing, 17th informational supplement 2007 Wayne, PA: CLSI M100 S17

[B7] Beceiro A Fernández-Cuenca F Ribera A Martínez-Martínez L Pascual A Vila J Rodríguez-Baño J Cisneros JM Pachón J Bou G Spanish Group for Nosocomial Infection (GEIH) False extended-spectrum beta-lactamase detection in Acinetobacter spp. due to intrinsic susceptibility to clavulanic acid J Antimicrob Chemother 2008 61 301 308 18065824 10.1093/jac/dkm461

[B8] Adams-Haduch JM Paterson DL Sidjabat HE Pasculle AW Potoski BA Muto CA Harrison LH Doi Y Genetic basis of multidrug resistance in *Acinetobacter baumannii* clinical isolates at a Tertiary Medical Center in Pennsylvania Antimicrob Agents Chemother 2008 52 3837 3843 10.1128/AAC.00570-08 18725452 PMC2573138

[B9] Pokhrel BM Shrestha B Sharma AP A prospective study of adult lower respiratory tract infections at TUTH in Kathmandu J Inst Med 1997 19 30 36

[B10] Mishra SK Koirala J Pokhrel BM Status of multidrug resistance and extended spectrum beta–lactamase producing strains causing lower respiratory tract and urinary tract infections among patients attending Tribhuvan University Teaching Hospital J Nepal Assoc Med Lab Sci 2005 7 30

[B11] Tamang MD Dey S Makaju RK Jha BK Shivananda PG Bhramadatan KN Bacterial aetiology of lower respiratory tract infection in patients attending Manipal Teaching Hospital J Nepal Assoc Med Lab Sci 2005 7 15 19

[B12] Falagas ME Karveli EA Siempos II Vardakas KZ *Acinetobacter* infections: a growing threat for critically ill patients Epidemiol Infect 2008 136 1009 1019 17892629 10.1017/S0950268807009478 PMC2870905

[B13] Joshi SG Litake GM Ghole VS Niphadkar KB Fluoroquinolone resistance from a transferable plasmid in *Acinetobacter calcoaceticus* Ind J Pathol Microbiol 2004 47 593 594 16295408

[B14] Mezzatesta ML Trovato G Gona F Nicolosi VM Nicolosi D Carattoli A Fadda G Nicoletti G Stefani S In vitro activity of tigecycline and comparators against carbapenem-susceptible and resistant *Acinetobacter baumannii* clinical isolates in Italy Ann Clin Microbiol Antimicrob 2008 7 4 10.1186/1476-0711-7-4 18261233 PMC2275293

[B15] Bergogne-Berezin E Towner KJ *Acinetobacer* spp. as nosocomial pathogens: microbiological, clinical and epidemiological features Clin Microbiol Rev 1996 9 148 165 8964033 10.1128/cmr.9.2.148 PMC172888

[B16] Higgins PG Wisplinghoff H Stefanki D Seifert H In vitro activities of the ß-lactamase inhibitors clavulanic acid, sulbactam, and tazobactam alone or in combination with ß-lactams against epidemiologically characterized multidrug-resistant *Acinetobacter baumannii* strains Antimicrob Agents Chemother 2004 48 1586 1592 10.1128/AAC.48.5.1586-1592.2004 15105109 PMC400525

[B17] Ling TKW Ying CM Lee CC Liu ZK Comparison of antimicrobial resistance of *Acinetobacter baumannii* clinical isolates from Shanghai and Hong Kong Med Princ Pract 2005 14 338 341 10.1159/000086932 16103700

[B18] Peleg AY Potoski BA Rea R Adams J Sethi J Capitano B Husain S Kwak EJ Bhat SV Paterson DL Acinetobacter baumannii bloodstream infection while receiving tigecycline: a cautionary report J Antimicrob Chemother 2007 59 128 131 17082201 10.1093/jac/dkl441

[B19] Gupta E Mohanty S Sood S Dhawan B Das BK Kapil A Emerging resistance to carbapenems in a tertiary care hospital in north India Ind J Med Res 2006 124 95 98 16926463

[B20] Sinha M Srinivasa H Mechanisms of resistance to carbapenems in meropenem- resistant *Acinetobacter* isolates from clinical samples Ind J Med Microbiol 2007 25 121 125 10.4103/0255-0857.32717 17582181

[B21] Gupta V Datta P Chander J Prevalence of metallo -beta lactamase (MBL) producing *Pseudomonas* spp. and *Acinetobacter* spp. in a tertiary care hospital in India J Infect 2006 52 311 314 10.1016/j.jinf.2005.08.013 16213590

[B22] Levin AS Barone AA Penco J Santos MV Marinho IS Arruda EA Manrique EI Costa SF Intravenous colistin as therapy for nosocomial infections caused by multidrug-resistant *Pseudomonas aeruginosa* and *Acinetobacter baumannii* Clin Infect Dis 1999 28 1008 1011 10.1086/514732 10452626

[B23] Kumari N Mohapatra TM Singh YI Prevalence of Methicillin-resisant *Staphylococcus aureus* (MRSA) in a tertiary-care hospital in eastern Nepal J Nepal Med Assoc 2008 47 53 56 18709031

[B24] Pokhrel BM Joshi R Bacteriological study at TU Teaching Hospital, Kathmandu, Nepal J Inst Med 1993 15 217 221

[B25] Subedi S Brahmadathan KN Antimicrobial susceptibility patterns of clinical isolates of *Staphylococcus aureus* in Nepal Clin Microb Infect 2005 11 235 237 10.1111/j.1469-0691.2004.01056.x 15715723

[B26] Gonzalez C Rubio M Romero-Vivas J Gonzalez M Picazo JJ Bacteremic pneumonia due to *Staphylococcus aureus*: a comparison of disease caused by methicillin-resistant & methicillin-susceptible organisms Clin Infect Dis 1999 29 1171 1177 10.1086/313440 10524959

[B27] McClelland RS Fowler VG Jr Sanders LL Gottlieb G Kong LK Sexton DJ Schmader K Lanclos KD Corey R *Staphylococcus aureus* bacteremia among elderly vs younger adult patients: comparison of clinical features and mortality Arch Intern Med 1999 159 1244 1247 10.1001/archinte.159.11.1244 10371233

[B28] Mukae H Iwamoto M Takase T Mori N Ishino T Doutsu Y Kohno S Yamaguchi K Hirota M Hara K Isolation of methicillin-resistant *Staphylococcus aureus* (MRSA) from sputum and clinical features of bronchopulmonary infection due to MRSA during 4 years (1985–1988) Nihon Kyobu Shikkan Gakkai Zasshi 1990 28 320 329 2355700

[B29] Lo WT Lin WJ Tseng MH Lu JJ Lee SY Chu ML Wang CC Nasal carriage of single clone of community acquired methicillin-resistant *Staphylococcus aureus* among Kindergarten attendees in northern Taiwan BMC Infectious Dis 2007 7 51 10.1186/1471-2334-7-51 PMC1906787 17543109

[B30] Dar JA Thoker MA Khan JA Ali A Khan MA Rizwan M Bhat KH Dar MJ Ahmed N Ahmad S Molecular epidemiology of clinical and carrier strains of methicillin resistant *Staphylococcus aureus* (MRSA) in the hospital settings of north India Ann Clin Microbiol Antimicrob 2006 5 22 10.1186/1476-0711-5-22 16972997 PMC1592298

